# P-307. Antibiotic Exposure and Community Resilience Shape Colonization Resistance to Vancomycin-Resistant Enterococci: Effects of Prior Antiboitics on Colonization Re-emergence

**DOI:** 10.1093/ofid/ofae631.510

**Published:** 2025-01-29

**Authors:** Ben Treat, Joseph Hobeika, Ellie Margolis

**Affiliations:** St. Jude Children's Research Hospital, Memphis, Tennessee; St. Jude Children's Research Hospital, Memphis, Tennessee; St. Jude Children's Research Hospital, Memphis, Tennessee

## Abstract

**Background:**

For life-threatening healthcare-associated vancomycin-resistant enterococci (VRE), gut colonization is a prerequisite for infection and is not predicted by prior Vancomycin treatment, indicating other selective forces are important. Host characteristics, strain variation and other microbial selective forces determine whether a patient's microbiome resists or permits VRE colonization.

**Figure:**
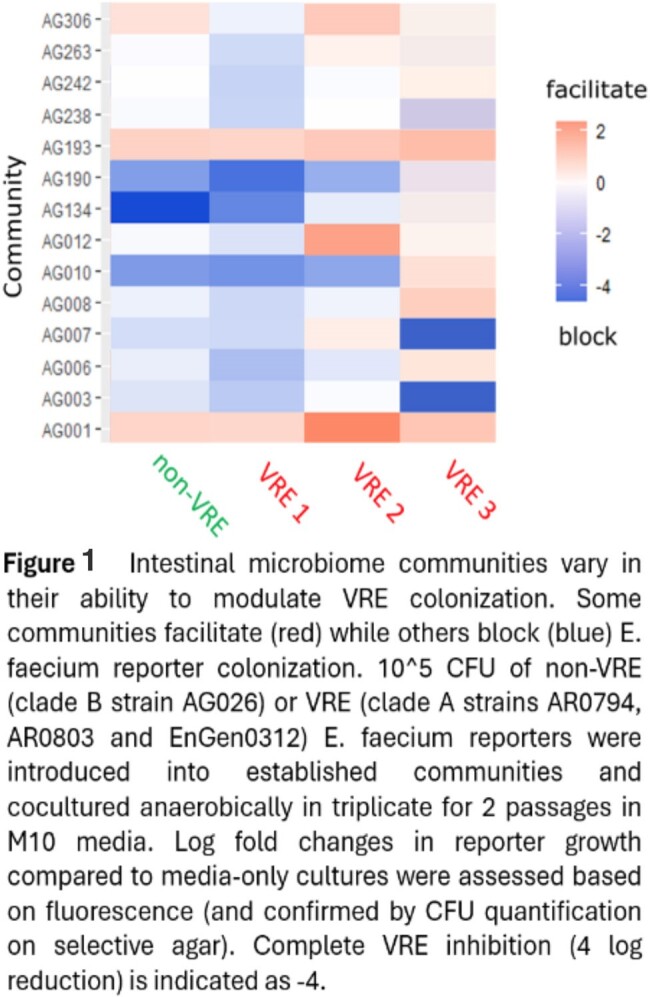

**Methods:**

Two preclinical models study VRE colonization resistance using fluorescently labeled clinical VRE strains. First, human microbiome stool communities were anaerobically cultivated in defined media, resulted in reproducible complex communities retaining 72-89% of microbial members. They are challenged with clinical VRE strains (10^3 cfu) to assess microbiome's role in resistance. Second, BL6 mice were pre-treated with PO Vancomycin, IP Cefepime+ IP Vancomycin, IP Meropenem, IP Pipercillin/Tazobactam, PO Metronidazole, or PO Clindamycin for one week. Post-treatment, they are inoculated with clinical VRE and clearance time was monitored. After a week without VRE detection in stool culture and by FACs, mice resume PO Vancomycin to observe re-emergence.

**Results:**

When complex human stool cultivated microbiomes are exposed to clinical VRE strains, some prevent colonization while others facilitate it. Intriguingly, both the VRE strain and the community composition influence colonization (fig 1). In mice treated with anaerobic-active antibiotics, consistent VRE colonization occured. However, duration varied significantly (e.g. PO Vancomycin cleared in 14-22 days, IP Pipercillin/Tazobactam in 20-54 days). After VRE clearance, re-challenge with antibiotics lead to VRE re-emergence in all previously colonized animals.

**Conclusion:**

Within human microbiome communities there is redundancy and variation in the members that can create a bacterial community barrier to VRE colonization. In mice, previous antibiotic use affects both how long VRE colonizes and its likelihood of returning after further disturbance. This may explain varying findings in studies on factors influencing MDRO colonization.

**Disclosures:**

**Ellie Margolis, MD PhD**, ICLR: Stocks/Bonds (Private Company)

